# Supplementary material for: The avifauna of Ramanathapuram, Tamil Nadu along the Southeast coast of India: waterbird assessments and conservation implications across key sanctuaries and Ramsar sites
Source: PeerJ. 2025 Feb 25;13:e18899. doi: 10.7717/peerj.18899 (PMC11869888; doi:10.7717/peerj.18899)
Supplement: Supplemental Information 2 [file peerj-13-18899-s002.docx]

|  | **CBS** | **KBS** | **MKBS** | **SBS** | **TBS** |
| --- | --- | --- | --- | --- | --- |
| **Dominance Index _D** | 0.1216 | 0.1127 | 0.1034 | 0.1718 | 0.1718 |
| **Shannon-Weiner Index_H** | 2.292 | 2.391 | 2.544 | 2.3 | 2.233 |
| **Pielou’s Evenness Index** | 0.761 | 0.7804 | 0.6702 | 0.587 | 0.4056 |
| **Menhinick Index** | 0.6183 | 0.6689 | 0.8372 | 0.3138 | 0.3138 |
