# Supplementary material for: The avifauna of Ramanathapuram, Tamil Nadu along the Southeast coast of India: waterbird assessments and conservation implications across key sanctuaries and Ramsar sites
Source: PeerJ. 2025 Feb 25;13:e18899. doi: 10.7717/peerj.18899 (PMC11869888; doi:10.7717/peerj.18899)
Supplement: Supplemental Information 3 — Threat ranking of various threat categories in the sanctuaries. [file peerj-13-18899-s003.docx]

|  | **Severity Score** | **Scope Score** | **Irreversibility Score** | **Total** | **Final Threat ranking** |
| --- | --- | --- | --- | --- | --- |
| **Livestock grazing** | 3 | 2 | 1 | 11 | Low |
| **Firewood Collection** | 3 | 2 | 2 | 12 | Medium |
| **Recreation/other disturbance** | 3 | 2 | 2 | 12 | Medium |
| **Fishing** | 2 | 2 | 3 | 11 | Low |
| **Feral Dogs** | 2 | 2 | 2 | 10 | Low |
| **Water Unavailability** | 5 | 5 | 3 | 23 | High |
| **Nesting Tree Unavailability** | 5 | 4 | 4 | 22 | High |
| **Invasive species** | 3 | 2 | 1 | 11 | Low |

**Table S2: Threat ranking of various threat categories in the sanctuaries**
